# Circular RNA_0057209 Acts as ceRNA to Inhibit Thyroid Cancer Progression by Promoting the STK4-Mediated Hippo Pathway *via* Sponging MicroRNA-183

**DOI:** 10.1155/2022/9974639

**Published:** 2022-03-11

**Authors:** Xinzhi Peng, Yue Zhu, Shaojian Lin, Weiming Yu, Cheng Zhang, Langping Tan, Miaoyun Long, Dingyuan Luo, Chengcheng Ji

**Affiliations:** ^1^Department of Thyroid Surgery, The Sun Yat-Sen Memorial Hospital, Sun Yat-Sen University, Guangzhou 510120, China; ^2^Department of Cardiology, The First Affiliated Hospital of Sun Yat-Sen University, Guangzhou 510080, China

## Abstract

Thyroid cancer is the most common malignancy of the endocrine system, and its outcome remains unsatisfactory. In recent years, circular RNAs (circRNAs) have emerged as crucial regulators in cancers. In the current study, we aimed to investigate whether and how circRNA_0057209 functioned in thyroid cancer. Initial results revealed that circRNA_0057209 and STK4 were both reduced, while miR-183 was up-regulated in thyroid cancer tissues and cells. Experiments including RNA pull-down and RIP assays further identified that upregulation of circRNA_0057209 augmented the expression of STK4, a target gene of miR-183, by competitively-binding to miR-183. Furthermore, functional experiments provided evidence that overexpression of circRNA_0057209 not only inhibited the proliferative, migratory, and invasive properties of thyroid cancer cells while facilitating their apoptosis but also delayed tumor growth. Conversely, upregulation of miR-183 or silencing of STK4 reversed the changes induced by circRNA_0057209. Meanwhile, mechanistic experimentation demonstrated that circRNA_0057209 promoted STK4 expression by sponging miR-183, while STK4 enhanced YAP phosphorylation to mediate the Hippo pathway, thereby suppressing tumor progression. Altogether, our findings indicated that circRNA_0057209 may serve as a competing endogenous RNA of miR-183 to increase STK4 expression, thus inhibiting the development of thyroid cancer.

## 1. Introduction

Thyroid cancer is the leading malignancy of the endocrine system, characterized by thyroid nodules and hyperthyroidism, where differentiated thyroid cancer is the most frequent subtype [1]. Unfortunately, the incidence of thyroid cancer has been increasing rapidly in recent decades [2], which has aroused major concerns for medical and health infrastructure bodies across the globe. A vast majority of thyroid cancer patients present with great prognosis; however, cases of locally invasive and/or distant metastatic thyroid cancer are featured by poor outcomes [3] and further lack satisfactory therapeutic regimens. Thus, it is of great important to further elucidate the pathogenesis of thyroid cancer to better tackle the endocrine malignancy.

Circular RNAs (circRNAs), a newly recognized class of noncoding RNA, have recently been highlighted as promising biomarkers and therapeutic targets for human diseases, owing to their critical crucial roles in the pathogenesis and progression of various tumors [4]. What is more, several circRNAs have been indicated to promote or suppress cell progression by interacting with microRNAs (miRNAs) or mediating certain pathways in the context of thyroid cancer, such as circRNA_ITCH [5]. In addition, a recent study indicated that hsa-circRNA_0011385 can accelerate the development of thyroid cancer by targeting miR-361-3p [6], which is suggestive of the critical interaction between circRNAs and miRNAs in the regulation of thyroid cancer progression. Furthermore, a large proportion of circRNAs possess at least one microRNA (miRNA) binding site and serve as competitive endogenous RNAs (ceRNAs) of miRNAs, thus regulating the expression of downstream target genes of miRNAs [7]. Nevertheless, the mechanism underlying the tumor-promoting or tumor-inhibiting action of circNRAs remains elusive and requires more in-depth investigation.

Moreover, deregulated expressions of a number of miRNAs have been previously associated with certain genetic lesions in thyroid cancer, which highlights the importance of miRNA analyses in the plasma for diagnosis [8]. For instance, intensified expression of miR-183-5p is known to enhance the viability, migrating, and invading phenotypes of thyroid carcinoma cells [9]. Moreover, reexpression of miR-183 was previously shown to aggravate thyroid cancer through negative regulation of PDCD4 protein expression at a posttranscriptional level [10]. Herein, we focused our efforts at elucidating the potential correlation between miR-183 and circRNAs in thyroid cancer progression.

Aside from the aforementioned circRNAs and miRNAs, the Hippo pathway is unraveled to be implicated in disease development. Furthermore, a prior study indicated a crucial role of this pathway in the pathogenesis of cancer, and in the invasion and metastasis of cancer [11]. Yes-activated protein-1 (YAP-1), a known protein of Hippo pathway, is regarded as a high-risk clinicopathologic prognosticator in papillary thyroid cancer [12]. Actually, the Hippo pathway is also engaged in regulating the biological processes such as tissue homeostasis and regeneration [13]. Interestingly, sterile 20-like family kinases serine/threonine protein kinase 4 (STK4), also referred to as mammalian sterile 20-like protein (MST1), is one of the kinase cascades of the Hippo pathway, while inhibition of miR-18a can activate the STK4-Hippo pathway, thus delaying the cervical cancer cell proliferation [14]. Besides, promotion of methylation of STK4 was previously shown to augment the apoptosis and autophagy of thyroid carcinoma cells [15]. To sum up, the interactions among circRNAs, miRNAs, and the Hippo pathway are crucial to the progression of cancer; however, their role still remains unknown with regard to thyroid cancer. As a result, the current study sets out to perform a series of *in vitro* and *in vivo* assays to explore the relationship among circRNA_0057209, miR-183, STK4, and Hippo pathway and their implications in thyroid tumorigenesis, in an effort to identify novel diagnostic and therapeutic targets against thyroid cancer.

## 2. Materials and Methods

### 2.1. Ethics Statement

The current study was approved by the Ethics Committee of The Sun Yat-Sen Memorial Hospital, Sun Yat-Sen University, and performed in strict compliance with the *Declaration of Helsinki*. Signed consents were attained from all participants and/or legal guardians prior to sample collection. Animal experiments were approved by the Animal Ethics Committee of The Sun Yat-Sen Memorial Hospital, Sun Yat-Sen University.

### 2.2. In Silico Analysis

Firstly, the circRNA expression dataset GSE93522 and the miRNA expression dataset GSE40807 related to thyroid cancer were both retrieved from the GEO database. The specific grouping of the samples in the expression dataset is shown in Supplementary Table 1. Differential analysis of the GSE93522 and GSE40807 datasets was subsequently performed using the R language “limma” package with ∣log2FC | >1 and *p* < 0.05 as the screening criteria. A heat map of the expression of differential circRNAs was then with the R language “pheatmap” package. Meanwhile, circRNA ID was converted to circBase ID with the help of the circBase database. In addition, miRNAs with potential binding sites for circRNAs were predicted using the circBank and CircInteractome databases. Afterwards, the miRNAs predicted by the circBank database were then intersected with the differentially expressed miRNAs in the GSE40807 dataset by means of a Venn diagram. The binding sites between circRNA and miRNA were further predicted utilizing the CircInteractome database. Additionally, bioinformatics tools miRWalk (binding probability≧1) and starBase were adopted to predict the potential target genes of miRNA. Intersection of miRNA candidate target genes and the downregulated genes in the GSE16544 dataset was obtained by means of a Venn diagram. Furthermore, the Metascape tool was employed to analyze the involved Gene Ontology biological processes of candidate target genes, while the starBase database was adopted to predict the miRNA binding site in the candidate target gene.

### 2.3. Patients and Tissue Samples

Paired thyroid cancer tissues and adjacent noncancerous tissues were excised from a total of 68 thyroid cancer patients at the Sun Yat-Sen Memorial Hospital, Sun Yat-Sen University, from August 2017 to August 2019, and immediately stored in sterile tubes in liquid nitrogen [16] for subsequent experimentation. All the patients presented with complete clinic pathological data and were pathologically diagnosed as thyroid cancer after surgery, while none of them underwent preoperative antitumor treatment.

### 2.4. Cell Culture

Four thyroid cancer cell lines (BCPAP, TPC-1, IHH-4, and HTH83) and one human normal thyroid cell line (NTHY-ORI3.1) were procured from BeNa Culture Collection (Beijing, China). The obtained cell lines were cultured in Dulbecco's modified Eagle's medium (DMEM) replenished with 10% fetal bovine serum and placed in a constant temperature incubator at 37°C with 5% CO_2_ in air. Subsequently, the cells were digested with 0.25% trypsin (HyClone Company, Logan, UT) after adhesion and those at the logarithmic phase of growth were collected for subsequent experiments. Additionally, TPC-1 and HTH83 cells were submitted to manipulation with overexpression- (oe-) circRNA_0057209 plasmid and miR-183 mimic as well as their corresponding controls, conforming to the specifications of Lipofectamine 2000 transfection reagent (Invitrogen Inc., Carlsbad, CA).

### 2.5. Quantitative Reverse Transcription Polymerase Chain Reaction (qRT-PCR)

Total RNA content was extracted from tissues or cells with assistance of the TRIzol reagent (Invitrogen). Subsequently, circRNA/mRNA and miRNA were reverse-transcribed with the help of PrimeScript RT kits (Takara, Tokyo, Japan) and miRNA first-strand synthesis kits (Takara), respectively. Next, the RNA (5 *μ*g) was incubated in 1.5 *μ*L RNase R (30 U) and 3 *μ*L 10x RNase R buffer. Meanwhile, circRNA_0057209, miR-183, STK4, and TTN-AS1 primers were designed using the Primer 5.0 software in accordance with the GenBank database and synthesized by Sangon Biotechnology (Shanghai, China). A 3500 reverse transcription system was adopted to perform reverse transcription PCR of total RNA. With glyceraldehyde-3-phosphate dehydrogenase (GAPDH) serving as the internal reference, the relative quantification method (2^-∆∆Ct^ method) was employed to calculate the relative expression of target genes. Each experiment was repeated three times independently to obtain the mean value.

**Figure 1:**
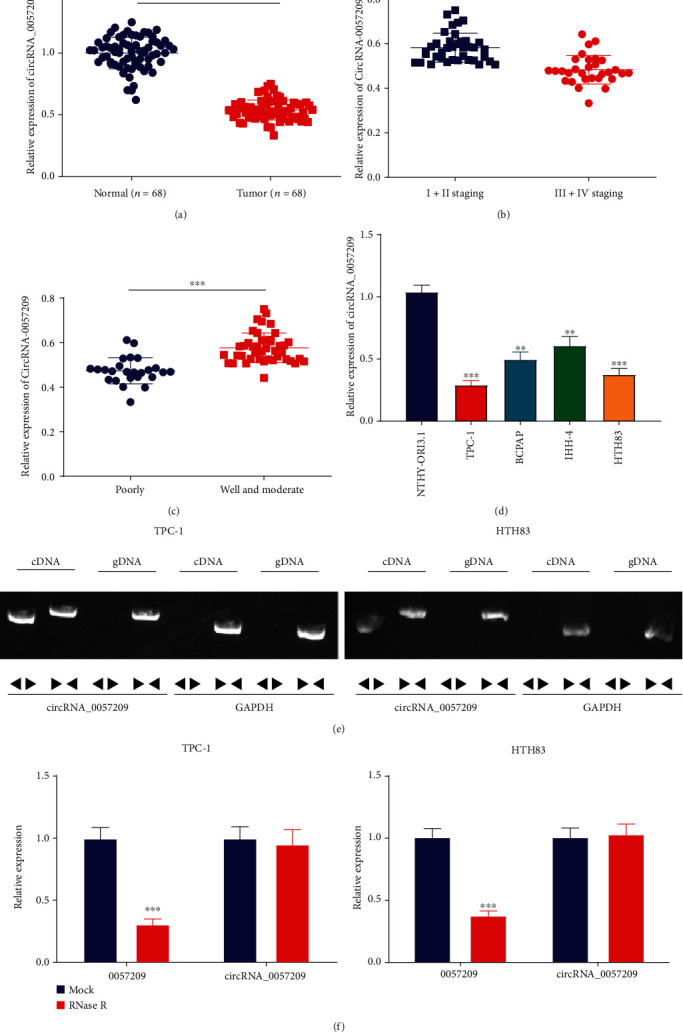
circRNA_0057209 expression was reduced in thyroid cancer tissues and cells. (a) The expression of circRNA_0057209 in thyroid cancer tissues and adjacent normal tissues determined by qRT-PCR. *n* = 68. ^∗^Compared with adjacent normal tissues, ^∗^*p* < 0.05. (b) The expression of circRNA_0057209 in thyroid cancer tissues of thyroid cancer patients at different TNM stages (*n* = 38 for I-II stage and *n* = 30 for III+IV stage). ^∗^Compared with the I+II staging group, *p* < 0.05, ^∗∗^*p* < 0.01, ^∗∗∗^*p* < 0.001. (c) circRNA_0057209 expression in thyroid cancer tissues of thyroid cancer patients with different differentiation degrees (*n* = 25 for poor differentiation and *n* = 43 for well and moderate differentiation). ^∗^Compared with the poor differentiation group, *p* < 0.05, ^∗∗^*p* < 0.01, ^∗∗∗^*p* < 0.001. (d) circRNA_0057209 expression in 4 kinds of thyroid cancer cells and normal thyroid cells determined by qRT-PCR. ^∗^Compared with NTHY-ORI3.1 cells, *p* < 0.05, ^∗∗^*p* < 0.01, ^∗∗∗^*p* < 0.001. (e) The amplification of circRNA_0057209 in cDNA and gDNA in TPC-1 and HTH83 cell lines determined by qRT-PCR. (f) mRNA abundance of circRNA_0057209 and linear 0057209 in TPC-1 and HTH83 cells treated with RNase R determined by qRT-PCR. ^∗^Compared with the NC group, *p* < 0.05, ^∗∗^*p* < 0.01, ^∗∗∗^*p* < 0.001. The cell experiment was repeated three times independently.

Additionally, for the identification of circRNA_0057209, the reverse-transcribed cDNA from RNA extracted from cells and genomic DNA (gDNA) extracted from cells were utilized as templates. According to the circRNA_0057209 structure and sequence, divergent primer pairs (circ-F/R) and convergent primer pairs (TTN-AS1-L-F/R) (primer sequences listed in Supplementary Table 2 and primer position shown in Supplementary Figure 1A) were designed, respectively. circRNA_0057209 was produced by ligating exon2 and exon11 of the TTN-AS1 gene (Supplementary Figure 1B, 1C). Simultaneously, circ-F was designed in exon11 and circ-R was designed in exon2, with the product size about 120 bp. Convergent primer pairs (TTN-AS1-L-F/R) were directly designed in exon5 of TTN-AS1 gene, with the product size about 320 bp. GAPDH was employed as the internal control (Figure 1(e)). In addition, TTN-AS1-TR-F/R primers were adopted for qRT-PCR determination of the linear expression patterns of TTN-AS1, and circ-F/R was utilized for qRT-PCR determination of the expression patterns of circRNA_0057209.

### 2.6. Western Blot Assay

Total protein content was extracted from tissues and cells, with protein concentration detected using bicinchoninic acid kits (20201ES76, Yeasen Company, Shanghai, China). The obtained proteins were subsequently by sodium dodecyl sulfate-polyacrylamide gel electrophoresis and electrotransferred onto a polyvinylidene fluoride membrane (IPVH85R, Millipore, Darmstadt, Germany). Next, the membrane was blocked with 5% bovine serum albumin at ambient temperature for 1 h and then submitted to overnight incubation at 4°C with the following diluted primary antibodies: STK4 (ab51134, 1 : 1000, Abcam Inc., Cambridge, UK), Bax (ab32503, 1 : 1000, Abcam), Bcl-2 (ab32124, 1 : 1000, Abcam), and GAPDH (ab9485, 1 : 2500, Abcam). After 3 TBST washes, 5 min each time, the membrane was incubated with horseradish peroxidase-labeled secondary antibody goat anti-rabbit IgG (ab97051, 1 : 20000, Abcam) for 1 h at room temperature. Enhanced chemiluminescence reagent (BB-3501, Amersham, Little Chalfont, UK) was adopted to visualize the protein bands which were photographed using the ChemiDoc XRS+imaging system (Bio-Rad, Hercules, CA). Finally, relative expression of each protein was normalized to GAPDH. The experiment was repeated three times independently to obtain the mean value.

### 2.7. Cell Counting Kit-8 (CCK-8) Assay

In line with the help of CCK-8 assay kits (CK04, Dojindo, Japan), cells were seeded in a 96-well plate at a density of 1 × 10^4^ per well and then precultured for 24 h. Next, the cells were transfected as described above for 48 h. At the 0 h, 24 h, 48 h, and 72 h time intervals after transfection, 10 *μ*L CCK-8 reagent was loaded for cellular incubation at 37°C for 2 h. Thereafter, the optical density (OD) value of each well at 450 nm was measured with the use of a microplate reader, and a cell growth curve was subsequently plotted.

### 2.8. Transwell Assay

Transwell chamber (pore size of 8 mm; 3452, Nanjing Xunbei Biotechnology Co., Ltd., Nanjing, China) without (for cell migration assay) or with Matrigel (66540030-1VL, Sigma-Aldrich Chemical Company, St. Louis, MO; for cell invasion assay) was employed for this assay. Each well of the upper chamber was filled with 300 *μ*L of serum-free DMEM, which contained the transfected cells (1 × 10^5^/mL), while the lower chamber was replenished with 700 *μ*L of complete medium. After incubation for 48 h, the cells were subjected to fixation in 4% paraformaldehyde, staining with crystal violet, and then photographing under an optical microscope (CX41-12C02, Olympus, Tokyo, Japan) in five randomly selected visual fields. Repeated experiments were conducted three times independently for calculating the mean value of cell count.

### 2.9. Flow Cytometry (FCM)

A total of 1 × 10^6^ cells were rinsed twice with phosphate-buffered saline (PBS) and then fixed in 70% cold ethanol. Next, the cells were mixed with 1 mL PI staining solution (50 *μ*g/mL, Becton, Dickinson and Company, Franklin Lakes, NJ) in conditions void of light for 30 min. Cell cycle was subsequently detected using a FACS Calibur flow cytometer (Becton, Dickinson and Company) and analyzed with the ModFit software.

For apoptosis detection, cells were suspended in 1× Annexin buffer in conditions void of light at ambient temperature for 10 min. Next, the cells were mixed with 5 *μ*L Annexin-VFITC (Becton, Dickinson and Company) and placed in a dark room at ambient temperature for 5 min. After PBS rinsing, the cells were re-suspended in 300 *μ*L 1× Annexin buffer, followed by FCM to detect the apoptosis rate. Repeated experimentation was implemented three times independently to calculate the mean value.

### 2.10. Fluorescence In Situ Hybridization (FISH)

FISH kits (Roche, Basel, Switzerland) were employed to detect the subcellular localization of circRNA_0057209. Following transfection, thyroid cancer TPC-1 cells were collected, rinsed twice with cold PBS, and then fixed with 4% paraformaldehyde. Next, the circRNA_0057209 was incubated with specific digoxigenin-labeled probe hybridization solution and the nucleus was stained with 4′,6-diamidino-2-phenylindole (DAPI) for 10 min at room temperature. Subsequently, the cells were rinsed twice with cold PBS and observed under a fluorescence microscope (BX53, Olympus). The mean value of three independent repeats was calculated.

### 2.11. Dual-Luciferase Reporter Gene Assay

The binding relationship of circRNA_0057209 or STK4 with miR-18 indicated by the biological website (https://cm.jefferson.edu/rna22/Interactive/) was verified by implementing a luciferase assay. Briefly, we analyzed the binding site and obtained the sequence of the binding site. Subsequently, the full length of circRNA_0057209 and the 3′UTR region of STK4 and the site-directed mutation sequence of circRNA_0057209 or STK4 with miR-18 binding site were cloned into the target sequence of the psiCheck2 plasmid downstream of the luciferase reporter gene. Afterward, cells were submitted to cotransfection of NC-mimic or miR-183 mimic with the luciferase reporter vector. After 48 h of incubation, the cells were lysed with 1× Passive lysis. The Firefly luciferase activity was then measured adopting a Dual Luciferase Reporter Assay System (E1910, Promega, Madison, WI) and luciferase assay kits (E2610, Promega, Madison, WI) with Renilla luciferase activity serving as the internal reference. Repeated experimentation was implemented three times independently to calculate the mean value.

### 2.12. RNA Binding Protein Immunoprecipitation (RIP)

RIP detection was performed with the help of Magna RIP kits (17-10499-2, Millipore, Billerica, MA). After 48 h of transfection, the TPC-1 cells were lysed with RIP lysis buffer. Next, incubation was conducted with Ago2 antibody or IgG antibody coupled magnetic beads. After purification, the enrichment of circRNA_0057209 and miR-183 was detected utilizing qRT-PCR.

### 2.13. RNA Pull-Down Assay

In brief, biotin-labeled miR-183 probe (bio-miR-183) or Bio-NC procured from Sangon was incubated with magnetic beads at 4°C overnight, after which the cells were lysed and collected. The magnetic bead mixture was utilized for 1 h incubation at ambient temperature, followed by purification and detection of circRNA_0057209 enrichment utilizing qRT-PCR.

### 2.14. Immunofluorescence Staining

After routine isolation and transfection, the cell culture (2 × 10^5^ cells/well) was conducted in an immunofluorescence chamber. Upon achieving 90% confluence, the cells were rinsed thrice with PBS on ice and fixed in 4% paraformaldehyde (1 mL/well) for 15 min. Next, the cells were treated with 0.3% Triton for 10 min and rinsed thrice with PBS, followed by sealing with goat serum for 30 min. The cells were then probed with primary antibody against YAP1 (ab52771, 1 : 200, Abcam) at 4°C overnight. The following day, cells were reprobed with goat anti-rabbit IgG (ab97051, 1 : 2000, Abcam) for 1 h at room temperature in the dark. Subsequently, DAPI was added to stain the nuclei within 15 min. Cells were rinsed thrice with PBS and sealed with a fluorescence quencher. Afterwards, observation and photographing were made under a fluorescence microscope (BX53, Olympus). Each experiment was repeated three times independently to obtain the mean value.

### 2.15. Immunohistochemistry (IHC)

The paraffin-embedded sections were dewaxed, dehydrated, and washed under tap water for 2 min. After reaction with 3% methanol-H_2_O_2_ for 20 min, the sections were rinsed with distilled water for 2 min and treated with 0.1 M PBS for 3 min, followed by antigen retrieval. Thereafter, the sections were blocked utilizing normal goat serum (C-0005, Shanghai Haoran Biotechnology Co., Ltd., Shanghai, China) and allowed to maintain at ambient temperature for 20 min. After that, the sections were probed with primary antibodies against STK4 (1 : 150, ab51134, Abcam) and p-YAP1 (ab76252, 1 : 200, Abcam) overnight at 4°C, and then re-probed with biotin-labeled goat anti-rabbit IgG (ab97051, dilution ratio of 1 : 2000, Abcam) at room temperature for 40 min. Subsequently, the samples were stained with 3,3′-diaminobenzidine (DA1010, Solarbio, Beijing, China) for 10 min, followed by 1 min counter-staining by hematoxylin (H8070, Solarbio), dehydration by gradient alcohol, clearing with xylene and mounting with neutral gum. The primary antibody was substituted with PBS as NC. Finally, the sections were observed under an optical microscope (CX41-12C02, Olympus) in 5 randomly selected high-power visual fields. The positive cells were those presenting with brown yellow granules, and the percentage of positive cell was calculated by 2 people blinded to the study.

### 2.16. Xenograft Tumor Model

A total of 36 female BALB/c nude mice (aged 3-4 week-old) were raised in a specific pathogen-free environment and grouped into the oe-circRNA_0057209+sh-NC, oe-circRNA_0057209+sh-STK4, and oe-NC+sh-NC groups. TPC-1 cells were transduced with corresponding lentivirus (pLVX-Puro-circRNA and pLVX-Puro-sh-RNA; Addgene) and the cells at the logarithmic phase of growth were prepared into a cell suspension with a concentration of 1 × 10^7^ cells/mL and then injected into the left axillary skin of nude mice with a 1 mL syringe. The long diameter (*A*) and short diameter (*B*) of mouse tumor were measured weekly using Vernier calipers for calculation of the tumor volume utilizing the formula described as follow: volume = (*A* × *B*^2^)/2. After 5 weeks of measurement, the nude mice were euthanized by decapitation after anesthesia, and the tumor tissues were extracted. Subsequently, the tumor was photographed, weighed, and measured. The expression patterns of STK4 and YAP1 phosphorylation levels were detected by means of IHC.

### 2.17. Statistical Analysis

SPSS 21.0 statistical software (IBM Corp., Armonk, NY) was adopted to statistically analyze all data. Measurement data were displayed as mean ± standard deviation. Data conforming to normal distribution and homogeneity of variance between two groups were compared using paired *t*-test, whereas unpaired *t*-test was utilized for data analysis between the other two groups. Data comparison among multiple groups were implemented using one-way analysis of variance (ANOVA), followed by Tukey's post hoc test, while that at different time points was made with repeated measures ANOVA and Bonferroni post hoc test. Pearson's correlation coefficient was employed for analyzing the correlation of the monitored indexes. A value of *p* < 0.05 was regarded statistically significant.

## 3. Results and Discussions

### 3.1. The Significance of circRNA_0057209, miR-183, and STK4 in Thyroid Cancer

**Figure 2:**
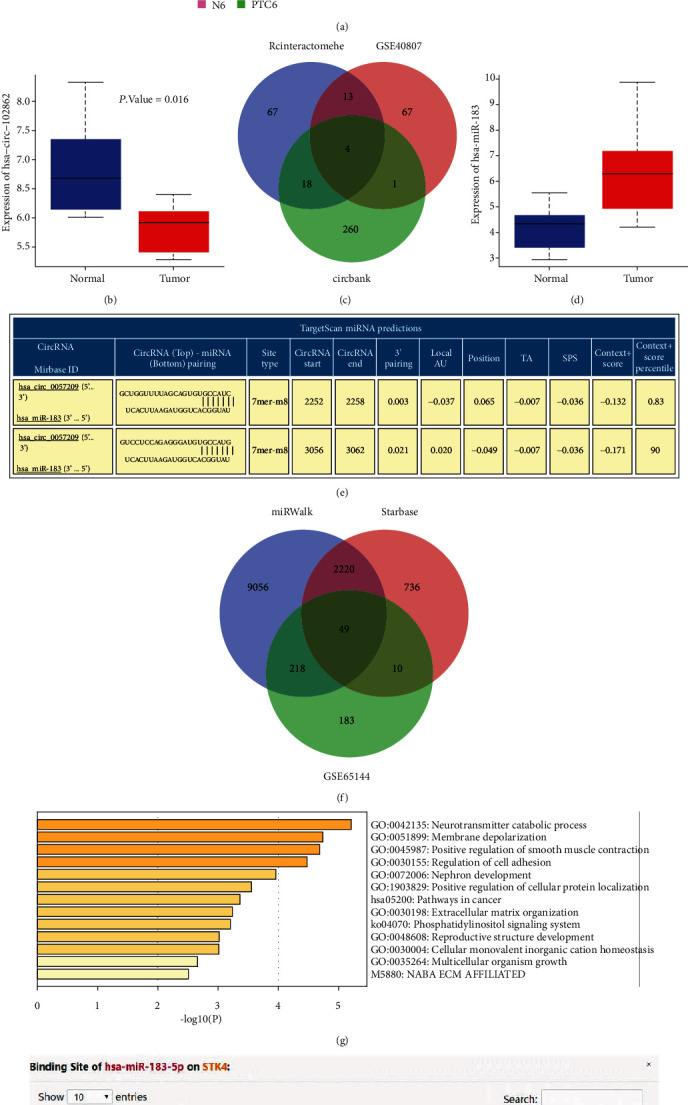
Bioinformatics analysis predicted the differentially expressed circRNAs, miRNAs, and mRNAs and their molecular interactions in thyroid cancer. (a) A heat map of the expression of the top 10 differentially expressed circRNAs with the smallest *p* value in the expression dataset GSE93522. The color scale from blue to red indicates that the expression value is from small to large. (b) circRNA_0057209 was lowly expressed in thyroid cancer. (c) Venn diagram of the miRNAs that bind to circRNA predicted by circBank and significantly upregulated miRNAs in the GSE40807 dataset. (d) hsa-miR-183 expression in thyroid cancer in the GSE40807 dataset. (e) The binding site between hsa_circ_0057209 and hsa-miR-183 predicted by CircInteractome. (f) Venn diagram of target genes of miRNA predicted by miRWalk and starBase and the significantly downregulated genes in the GSE65144 dataset. (g) Enrichment analysis results of candidate genes involved biological processes. (h) The binding site between hsa-miR-183 and STK4 predicted by starBase. (i) Correlation analysis between miR-183 and STK4 in thyroid cancer.

Initial differential analyses of the circRNA expression dataset GSE93522 related to thyroid cancer reared a total of 128 differentially expressed circRNAs, comprising of 108 significantly highly expressed circRNAs and 20 lowly expressed circRNAs, and the expression heat map of the top 10 differential circRNAs with the smallest *p* value was drawn (Figure 2(a)). Meanwhile, differential analyses of the GSE101123 dataset revealed that has_c_0057209 (alias hsa_circ_102862) was poorly expressed in thyroid cancer (Figure 2(b)). In order to predict the downstream miRNAs of hsa_circ_0057209, we further obtained 85 and 285 miRNAs with potential binding sites to the candidate circRNA through the CircInteractomehe and circBank databases, respectively. The intersection of the differentially expressed miRNAs in GSE40807 and the predicted miRNAs from circBank is shown in Figure 2(c), with hsa-miR-654-3p, hsa-miR-769-5p, hsa-miR-183, and hsa-miR-338-3p being at the intersection. Interestingly, previous studies have emphasized the correlation between hsa-miR-183 and thyroid cancer [10]. Subsequent differential analyses of the GSE40807 dataset demonstrated that hsa-miR-183 was significantly highly expressed in thyroid cancer (Figure 2(d)). The binding sites between hsa_circ_0057209 and hsa-miR-183 were also obtained from the CircInteractome database (Figure 2(e)). To further predict the downstream target genes of hsa-miR-183, 11543 and 3015 potential target genes of miRNA were predicted using the bioinformatics tool miRWalk and starBase databases, respectively, while 460 downregulated genes in GSE65144 were retrieved from the GEO database. The intersection of candidate target genes and thyroid cancer-related genes yielded a total of 49 genes at the intersection (Figure 2(f)). The Metascape tool was adopted to analyze the biological processes involved in the aforementioned 49 candidate target genes, which revealed that these genes were implicated in cancer pathways (Figure 2(g)). Moreover, the STK4 gene was previously documented to participate in the metastasis of thyroid cancer through the Hippo pathway [17]. The binding sites of hsa-miR-183 and STK4 were subsequently obtained from the starBase database (Figure 2(h)), while miR-183 and STK4 were found to exhibit a negative-correlation in thyroid cancer (Figure 2(i)). Altogether, these findings suggested that circRNA_0057209 may modulate the expression of STK4 by regulating miR-183, thereby affecting the Hippo pathway to participate in the progression of thyroid cancer.

### 3.2. circRNA_0057209 Was Poorly Expressed in Thyroid Cancer Tissues and Cells

Furthermore, we adopted qRT-PCR to determine the expression patterns of circRNA_0057209 in thyroid cancer cells and tissues, which revealed higher expression of circRNA_0057209 in thyroid cancer tissue (n = 68) compared with adjacent normal tissues (Figure 1(a)). Additionally, the relationship between the expression profile of circRNA_0057209 and the clinical indicators of patients with thyroid cancer were statistically analyzed, and the subsequent results uncovered a significant correlation of circRNA_0057209 expression with patient's tumor size, TNM stage, tumor differentiation, and lymph node metastasis (Supplementary Table 3). At the same time, qRT-PCR was employed again to determine the expression patterns of circRNA_0057209 in different TNM stages and different levels of differentiation in patients with thyroid cancer. It was found that circRNA_0057209 expression levels were up-regulated in stages III+IV or poorly differentiated thyroid cancer (Figures 1(b) and 1(c)). Additionally, compared to normal thyroid cell line NTHY-ORI3.1, circRNA_0057209 expression levels were found to considerably reduced in human thyroid cancer cell lines (BCPAP, TPC-1, IHH-4, and HTH83), with TPC-1 cells and HTH83 cells presenting with the lowest circRNA_0057209 expressions. As a result, TPC-1 cells and HTH83 cells were chosen for subsequent experimentation (Figure 1(d)). Moreover, in the genome, circRNA_0057209 was found to be transcribed from TTN-AS1, while exon2 and exon11 were linked to form a circular RNA during the mRNA splicing process (Supplementary Figure 1B, 1C). For verification, while cDNA and gDNA of TPC-1 and HTH83 cell lines were used as templates, cDNA was amplified to hsa_circ_0057209 only with amplification primers (Figure 1(e)). Subsequently, total RNA content collected from TPC-1 cells and HTH83 cells was digested with RNase R, and the expression of linear 0057209 was found to be remarkably decreased, while that of circRNA_0057209 was unaltered (Figure 1(f)), indicating that circRNA_0057209 may be more resistant to digestion than linear 0057209. Besides, the results of qRT-PCR illustrated lower expression levels of TTN-AS1-RNA in TPC-1 and HTH83 cells as compared to those of linear TTN-AS1-RNA expression (Supplementary Figure 1D). Collectively, these findings indicated the ectopic expression of circRNA_0057209 in both thyroid cancer tissues and cells.

### 3.3. Over-Expression of circRNA_0057209 Inhibited the Proliferation, Migration, and Invasion of Thyroid Cancer Cells but Promoted Apoptosis

**Figure 3:**
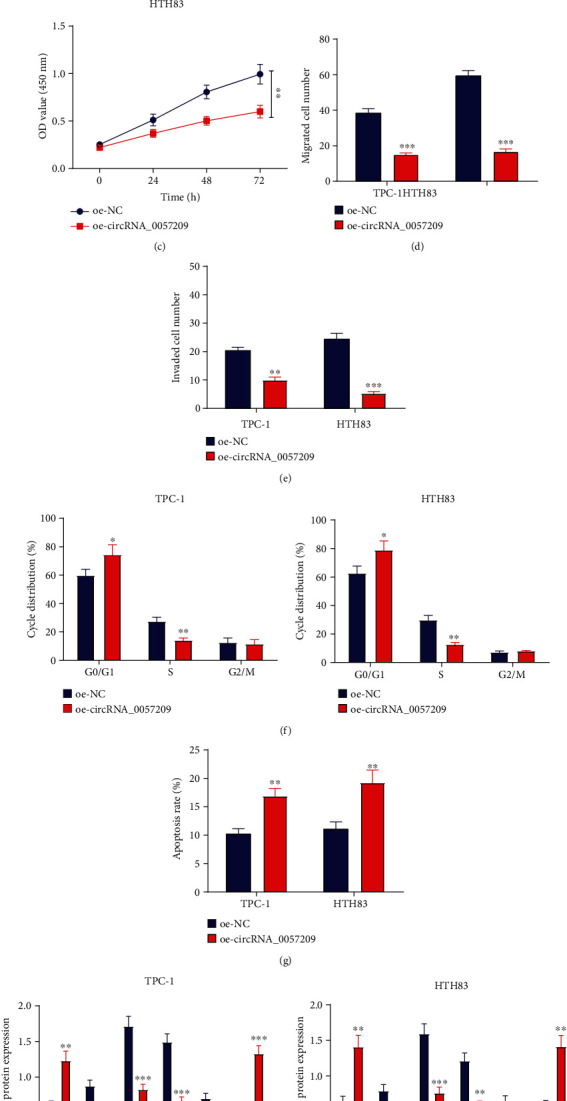
High expression of circRNA_0057209 inhibited the proliferation, migration, and invasion of thyroid cancer cells. (a) The relative expression of circRNA_0057209 in oe-circRNA_0057209-treated TPC-1 and HTH83 cells determined by qRT-PCR. (b) CCK-8 showing the proliferation of oe-circRNA_0057209-treated TPC-1 cells. (c) CCK-8 showing the proliferation of oe-circRNA_0057209-treated HTH83 cells. (d) Transwell assay showing the migration of oe-circRNA_0057209-treated TPC-1 and HTH83 cells. (e) Transwell assay showing the invasion of oe-circRNA_0057209-treated TPC-1 and HTH83 cells. (f) Cell cycle distribution upon circRNA_0057209 overexpression analyzed using FCM. (g) Apoptosis rate of oe-circRNA_0057209-treated TPC-1 and HTH83 cells assessed using FCM. (h) Western blot showing the relative expression of E-cadherin, N-cadherin, Ki67, PCNA, Bax, and Bcl-2 in oe-circRNA_0057209-treated TPC-1 and HTH83 cells. ^∗^ *p* < 0.05 compared with the oe-NC group. The cell experiment was repeated three times independently.

After uncovering the low expression patterns of circRNA_0057209 in thyroid cancer tissues and cell lines, we performed functional experiments in thyroid cancer cell lines TPC-1 and HTH83 to further investigate the effect of circRNA_0057209 on their biological functions. We employed plasmids expressing circRNA_0057209, whose transfection efficiency in cancer cells was assessed by means of qRT-PCR (Figure 3(a)). It was found that overexpression of circRNA_0057209 markedly attenuated the proliferative, migrating, and invasive abilities of thyroid cancer cells (Figures 3(b)–3(e), Supplementary Figure 2A–2B). Simultaneously, FCM was adopted to assess the cell cycle and apoptosis upon transfection, which revealed that the ratio of G0/G1 phase cells in the oe-circRNA_0057209-treated cells was significantly increased, while the S phase cells were profoundly reduced and the apoptotic ability was markedly enhanced (Figures 3(f) and 3(g)). In addition, we also performed a Western blot assay to determine the expression patterns of EMT-, proliferation- and apoptosis-related proteins in TPC-1 and HTH83 cells. Subsequent results demonstrated that the expression levels of E-cadherin and Bax were notably increased, while those of N-cadherin, Ki67, PCNA, and Bcl-2 were markedly decreased following treatment with oe-circRNA_0057209 (Figure 3(h)). Overall, these findings demonstrated that high expressions of circRNA_0057209 might inhibit the malignant properties of thyroid cancer cells.

### 3.4. circRNA_0057209 Competitively Bound to miR-183 to Reduce Its Expression

**Figure 4:**
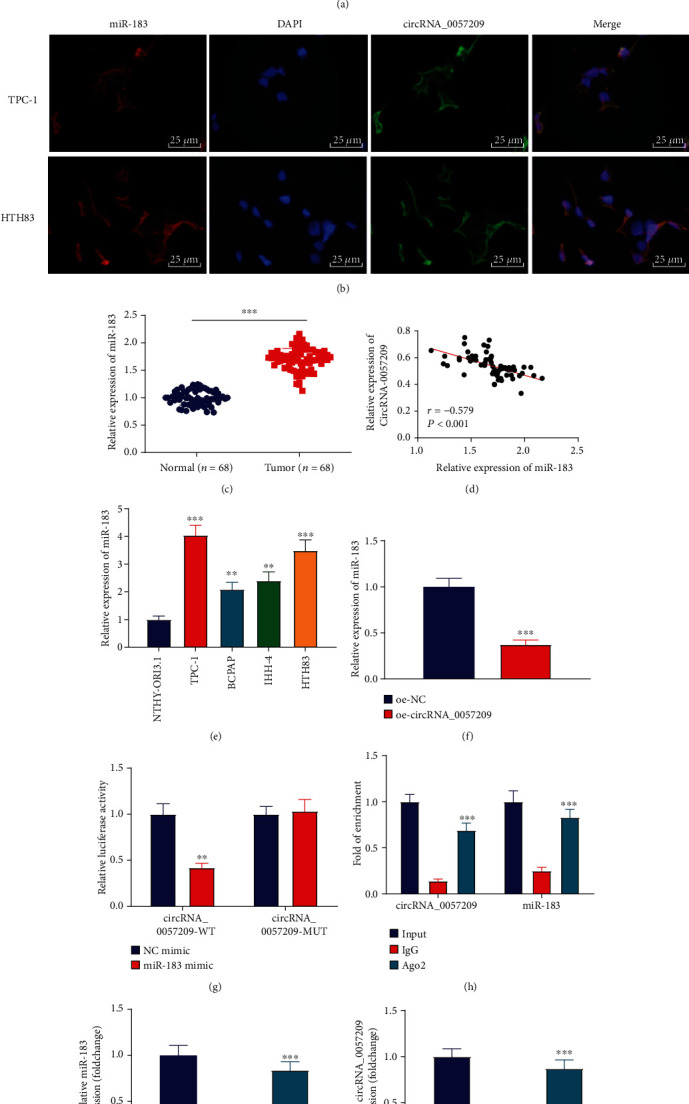
circRNA_0057209 competitively binds to miR-183 to reduce its expression in thyroid cancer cells. (a) Predicted binding sites of circRNA_0057209 and miR-183. (b) FISH analysis of circRNA_0057209 colocalization with miR-183 cells. The blue part in the figure represents the nucleus, the green part represents circRNA_0057209, and the red part represents miR-183 (×400). (c) The expression of miR-183 in thyroid cancer tissues and adjacent normal tissues determined by qRT-PCR, *n* = 68, *p* < 0.05, ^∗∗^*p* < 0.01, ^∗∗∗^*p* < 0.001. (d) Correlation analysis between circRNA_0057209 and miR-183. (e) The expression of miR-183 in four kinds of thyroid cancer cells and normal thyroid cells determined by qRT-PCR. ^∗^Compared with NTHY-ORI3.1 cells, *p* < 0.05, ^∗∗^*p* < 0.01, ^∗∗∗^*p* < 0.001. (f) The expression of miR-183 in cells treated with oe-circRNA_0057209 determined by qRT-PCR. ^∗^*p* < 0.05, ^∗∗^*p* < 0.01, ^∗∗∗^*p* < 0.001. (g) Dual luciferase reporter gene assay for identification of the interaction between circRNA_0057209 and miR-183 upon treatment with oe-NC or oe-circRNA_0057209. ^∗^Compared with the NC mimic group *p* < 0.05, ^∗∗^*p* < 0.01, ^∗∗∗^*p* < 0.001. (h) RIP assay determining the binding of circRNA_0057209, miR-183 and Ago2 or IgG. ^∗^Compared with the input group and ^#^ indicated compared with the IgG group, *p* < 0.05, ^∗∗^*p* < 0.01, ^∗∗∗^*p* < 0.001; ^##^*p* < 0.01, ^###^*p* < 0.001. (i) Interaction between miR-183 and circRNA_0057209 analyzed by RNA pull-down assay. ^∗^*p* < 0.05 compared with the NC-biotin group, ^∗∗^*p* < 0.01, ^∗∗∗^*p* < 0.001. The cell experiment was repeated three times independently.

To further investigate the underlying mechanism of circRNA_0057209, we conducted bioinformatics analysis using online prediction software (https://circinteractome.nia.nih.gov/) to detect its potential target miRNAs. It was found that the circRNA_0057209 sequence contained a complementary binding sequence for miR-183 (Figure 4(a)). FISH experimentation was subsequently performed for verification, and the results demonstrated the cytoplasmic co-localization of circRNA_0057209 and miR-183 (Figure 4(b)). In addition, we observed that miR-183 expression levels were upregulated in thyroid cancer tissues relative to adjacent normal tissues (Figure 4(c)). Meanwhile, a strong negative correlation was observed between circRNA_0057209 and miR-183 in thyroid cancer cells (Figure 4(d)). Furthermore, the results of qRT-PCR revealed that miR-183 expression levels were significantly upregulated in human thyroid cancer cell lines, especially in TPC-1 and HTH83 cells (Figure 4(e)). Upon further treatment with oe-circRNA_0057209, miR-183 expression was found to be significantly diminished (Figure 4(f)). On a separate note, results of dual luciferase reporter assay showed that transfection with miR-183 mimic reduced the luciferase activity of circRNA_0057209-WT without altering that of circRNA_0057209-MUT (Figure 4(g)), thus validating the interaction between circRNA_0057209 and miR-183. Moreover, RIP experimentation was carried out, which displayed that circRNA_0057209 and miR-183 bound by Ago2 were both significantly increased (Figure 4(h)). Subsequent RNA pull-down results additionally validated the interaction between circRNA_0057209 and miR-183. Furthermore, we incubated biotin-labeled miR-183 with magnetic beads and incubated the magnetic bead mixture with the cell lysate. By detecting the enrichment of circRNA_0057209 and miR-183, miR-183 was witnessed to specifically bind to circRNA_0057209 (Figure 4(i)). Altogether, these findings indicated that circRNA_0057209 can competitively-bind to miR-183 to reduce its expression.

### 3.5. Overexpression of miR-183 Reversed the Tumor-Suppressive Effect of circRNA_0057209

**Figure 5:**
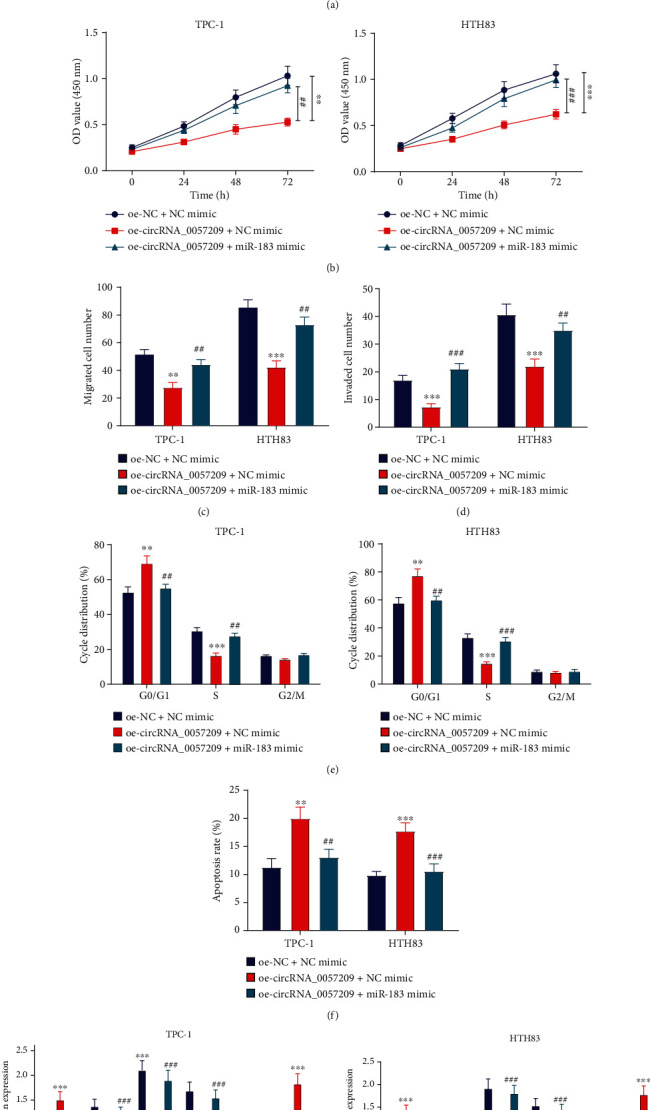
Upregulation of miR-183 abrogated the tumor-suppressive effect of circRNA_0057209 on thyroid cancer. Thyroid cancer cells were treated with oe-circRNA_0057209 or combined with miR-183 mimic. (a) The expression of circRNA_0057209 and miR-183 in thyroid cancer cells determined by qRT-PCR. (b) CCK-8 showing thyroid cancer cell proliferation. (c) Transwell assay showing thyroid cancer cell migration. (d) Transwell assay showing cell invasion. (e) FCM images of thyroid cancer cell cycle distribution. (f) FCM showing cell apoptosis rate. (g) Western blot assay showing the relative protein expression of E-cadherin, N-cadherin, Ki67, PCNA, Bax, and Bcl-2 in thyroid cancer cells. ^∗^Compared with the oe-NC+NC mimic group, ^#^ indicated that compared with the oe-circRNA_0057209+NC mimic group, *p* < 0.05, ^∗∗^*p* < 0.01, ^∗∗∗^*p* < 0.001; ^##^*p* < 0.01, ^###^*p* < 0.001. The cell experiment was repeated three times independently.

The aforementioned findings allowed us to examine whether circRNA_0057209 could influence the biological functions of thyroid cancer cells by regulating miR-183. In addition, the results of qRT-PCR demonstrated that oe-circRNA_0057209 could significantly elevate the expression of circRNA_0057209 and reduce that of miR-183. However, we found that additional application of miR-182 mimic hardly changed the expression of circRNA_0057209 and only elevated the miR-183 expression (Figure 5(a)). Meanwhile, treatment with oe-circRNA_0057209 retarded the proliferating, migrating, and invading capacities of thyroid cancer cells, while these capacities were restored in the presence of miR-183 mimic (Figures 5(b)–5(d), Supplementary Figure 2C-2D). At the same time, the ratio of G0/G1 phase cells in the oe-circRNA_0057209-treated cells was observed to be significantly increased, while the proportion of S phase cells was markedly reduced and the apoptotic ability was significantly enhanced. On the other hand, enhancement of the miR-183 expression exerted opposite effects to those of oe-circRNA, even restoring the cell cycle and apoptotic ability (Figures 5(e) and 5(f)). Furthermore, the results of Western blot assay indicated that E-cadherin and Bax expression levels were considerably upregulated in cells, while those of N-cadherin, Ki67, PCNA, and Bcl-2 were considerably down-regulated upon treatment with oe-circRNA_0057209, whereas the addition of miR-183 abrogated the effect of oe-circRNA_0057209 (Figure 5(g)). Collectively, high expression of miR-183 could reverse the inhibiting effect of circRNA_0057209 on thyroid cancer progression.

### 3.6. circRNA_0057209 Competitively Bound to miR-183 and Thus Upregulated the Expression of STK4

**Figure 6:**
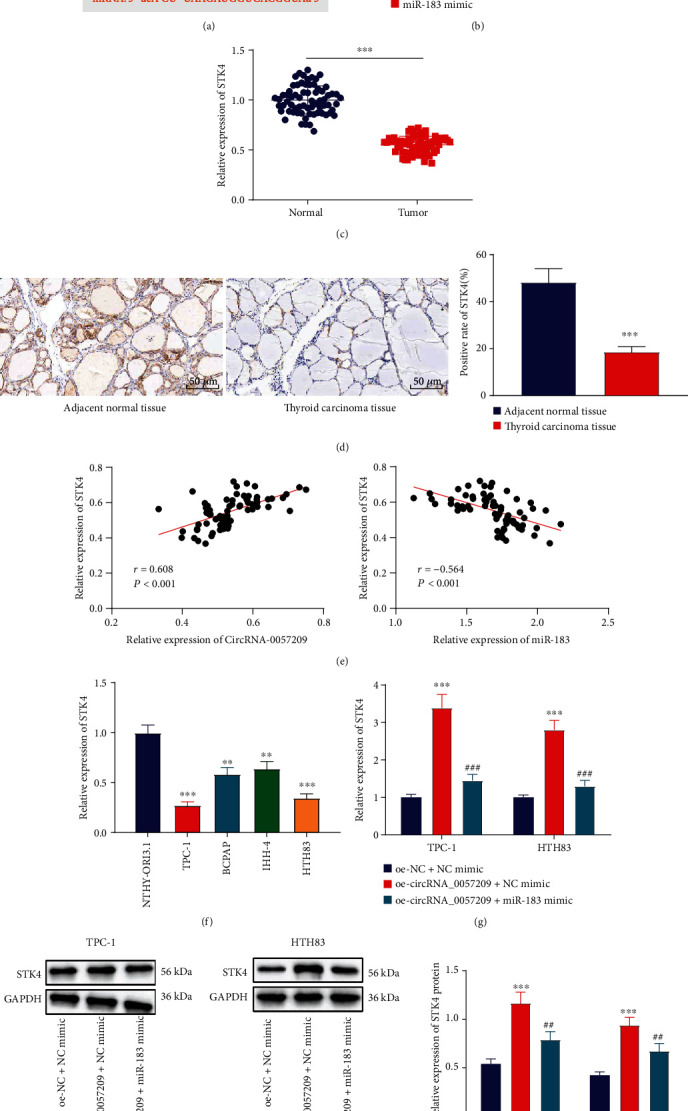
circRNA_0057209 acted as ceRNA of miR-183 to upregulate the expression of STK4 (a) The predicted binding site between miR-183 and STK4 by bioinformatics analysis. (b) Dual luciferase activity detection to verify the binding between miR-183 and STK4. (c) STK4 expression in thyroid cancer tissues and adjacent normal tissues determined by qRT-PCR. (d) Representative images of IHC of STK4-positive cells thyroid cancer tissues and adjacent normal tissues (×200). (e) Correlation analysis between circRNA_0057209 and STK4, miR-183, and STK4. (f) The expression of STK4 in BCPAP, TPC-1, IHH-4, HTH83, and NTHY-ORI3.1 cells determined by qRT-PCR. (g) The mRNA expression of STK4 in cells upon treatment with oe-circRNA_0057209 or combined with miR-183 mimic determined by qRT-PCR. (h) Western blot assay showing the relative protein expression of STK4 in cells upon treatment with oe-circRNA_0057209 or combined with miR-183 mimic. ^∗^Compared with the oe-NC+NC mimic group, *p* < 0.05, ^#^ indicated that compared with the oe-circRNA_0057209+NC mimic group, *p* < 0.05, ^∗∗^*p* < 0.01, ^∗∗∗^*p* < 0.001; ^##^*p* < 0.01, ^###^*p* < 0.001. The cell experiment was repeated three times independently.

The starBase database identified the presence of binding sites between STK4 and miR-183 (Figure 6(a)). Subsequently, a dual luciferase reporter assay was carried out, and the results of which confirmed that the luciferase activity of STK4-WT was significantly reduced upon treatment with miR-183 mimic, while that of STK4-MUT was unaltered (Figure 6(b)), indicating that miR-183 might target and bind to STK4.

Additionally, the results of qRT-PCR revealed that STK4 was poorly expressed in cancer tissues relative to adjacent normal tissues (Figure 6(c)). Meanwhile, IHC results illustrated the cytoplasmic positive expression of STK4 and remarkably lower positive expression levels of STK4 in thyroid cancer tissues versus those in adjacent non-cancerous tissues (Figure 6(d)). Moreover, a positive-relationship was documented between circRNA_0057209 and STK4, as well as a negative relationship between miR-183 and STK4 in thyroid cancer cells (Figure 6(e)). Consistently, downregulation of STK4 was observed in BCPAP, TPC-1, IHH-4, and HTH83 cells, with TPC-1 and HTH83 cells exhibiting the lowest SKT4 expression levels (Figure 6(f)). To further analyze whether circRNA_0057209 regulated STK4 through miR-183, qRT-PCR and Western blot analyses were subsequently implemented to characterize the STK4 expression patterns. It was found that the mRNA and protein expression levels of STK4 were significantly increased in TPC-1 and HTH83 cells following treatment with oe-circRNA_0057209, yet this increase could be countered by miR-183 mimic (Figures 6(g) and 6(h)). Overall, these findings suggested that circRNA_0057209 could elevate STK4 expression by competitively-binding to miR-183.

### 3.7. STK4 Suppressed the Proliferation, Migration, and Invasion of Thyroid Cancer Cells and Promoted Cell Apoptosis by Regulating the Hippo Pathway

**Figure 7:**
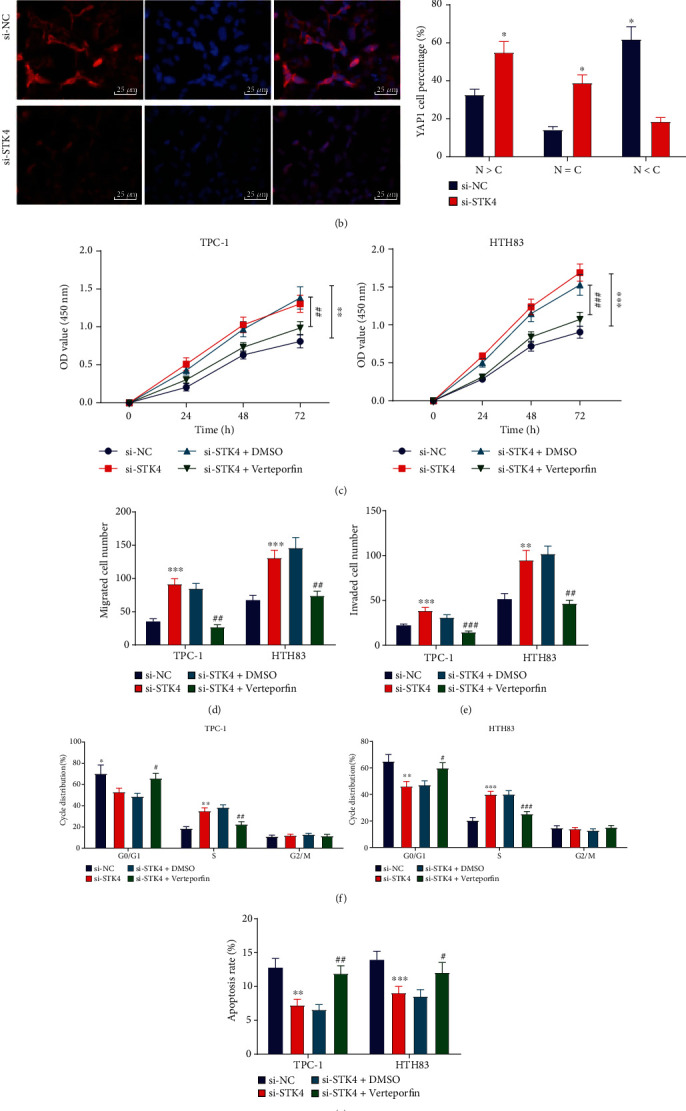
STK4 impairs the proliferation, migration, and invasion of thyroid cancer cells while inducing cell apoptosis by regulating the Hippo pathway. (a) Western blot assay displaying the protein expression of Hippo pathway-related factors (MST1, LAST1, LAST2, YAP1, and p-YAP1) in si-STK4-treated thyroid cancer cells. (b) Immunofluorescence staining displaying the location of YAP1 and the nuclear/cytoplasmic ratio (N/C) in si-STK4-treated thyroid cancer cells. Red represents YAP1 and blue represents the nucleus (×400). Thyroid cancer cells were treated with si-STK4 or combined with verteporfin. (c) CCK-8 displaying the thyroid cancer cell proliferation. (d) Transwell assay displaying the migration of thyroid cancer cells. (e) Transwell assay displaying the invasion of thyroid cancer cells. (f) FCM analysis of cell cycle distribution. (g) FCM displaying the cell apoptosis rate. (h) Western blot assay displaying the relative protein expression of E-cadherin, N-cadherin, Ki67, PCNA, Bax, and Bcl-2 in thyroid cancer cells. ^∗^Compared with the si-STK4 group, *p* < 0.05, # indicated compared with the si-STK4+DMSO group, *p* < 0.05, ^∗∗^*p* < 0.01, ^∗∗∗^*p* < 0.001; ^##^*p* < 0.01, ^###^*p* < 0.001. The cell experiment was repeated three times independently.

Existing evidence suggests that STK4 possesses the potential to regulate the Hippo pathway to stimulate the phosphorylation of YAP [17]. Consequently, we set out to determine whether STK4 could mediate the biological functions of thyroid cancer cells through the Hippo pathway. As expected, treatment with si-STK4 reduced the protein expression levels of MST1, LAST1, and LAST2, as well as the YAP1 phosphorylation levels in thyroid cancer cells (Figure 7(a)). Meanwhile, Immunofluorescence staining illustrated that YAP1 was primarily located in the nuclei of thyroid cancer cells following treatment with si-STK4 (Figure 7(b)). Besides, the results of CCK-8 and Transwell assays revealed that silencing of STK4 facilitated the proliferative, migratory, and invasive capabilities of thyroid cancer cells, whereas additional treatment with verteporfin (a YAP inhibitor, S1786, Selleck) countered these trends (Figures 7(c)–7(e)). At the same time, FCM data revealed that silencing of STK4 reduced the proportion of G0/G1-arrested cells and induced S phase-arrested cells, accompanied with decreased cell apoptosis ability. However, the addition of verteporfin reversed the changes induced by si-STK4, restoring the apoptotic ability (Figures 7(f) and 7(g)). Moreover, the results of Western blot assay indicated a reduction in the E-cadherin and Bax expression levels, yet an increase in those of N-cadherin, PCNA, and Bcl-2 in si-STK4-treated thyroid cancer cells. On the other hand, combined treatment with verteporfin and si-STK4 elevated the E-cadherin and Bax expression levels, but decreased those of N-cadherin, PCNA, and Bcl-2 compared with treatment of DMSO and si-STK4 (Figure 7(h)). Overall, these findings showed that STK4 could impair the malignant progression of thyroid cancer by regulating the Hippo pathway.

### 3.8. circRNA_0057209 Inhibited Thyroid Tumorigenesis by Regulating the miR-183/STK4 Signaling

**Figure 8:**
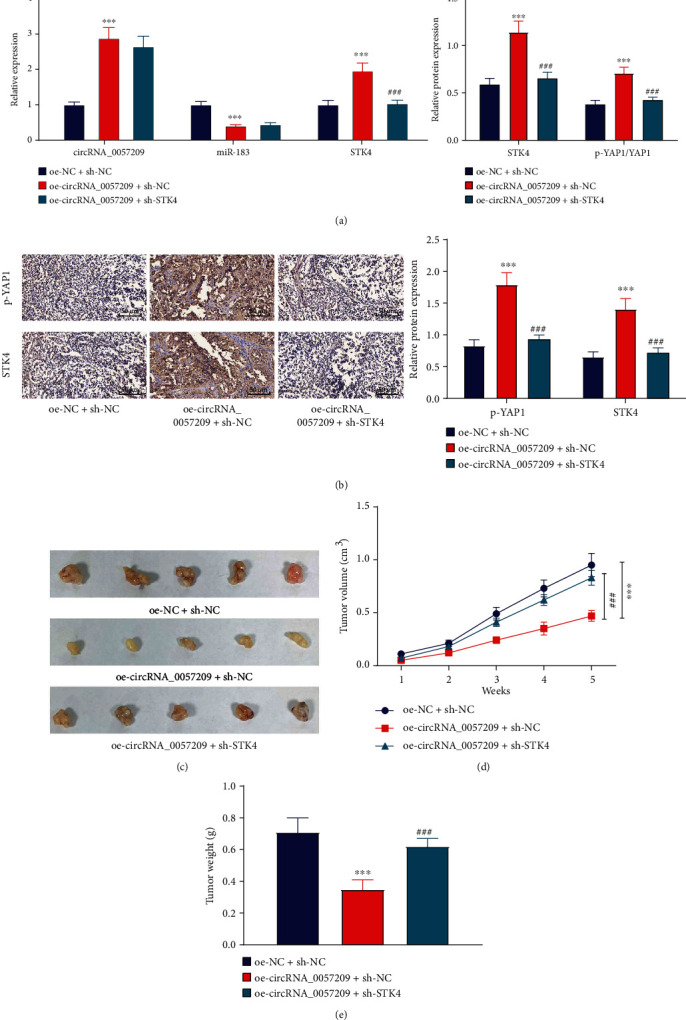
circRNA_0057209 arrested the tumorigenesis of thyroid cancer by regulating the miR-183/STK4 signaling. Nude mice were injected with TPC-1 cells transduced with lentivirus carrying oe-circRNA_0057209 or sh-STK4. (a) qRT-PCR and Western blot analyses showing the expression of circRNA_0057209, STK4 and miR-183, as well as YAP1 phosphorylation levels in tumor tissues of mice. (b) IHC results showing the YAP1 phosphorylation levels and STK4 protein expression in tumor tissues of mice (×200). (c) Representative macroscopic images of transplanted tumors in nude mice. (d) Quantification of tumor volume. (e) Weight of transplanted tumors in nude mice. ^∗^Compared with compared with the oe-NC+sh-NC group, *p* < 0.05, ^#^ indicated that compared with the oe-circRNA_0057209_0057209+sh-NC group, *p* < 0.05, ^∗∗^*p* < 0.01, ^∗∗∗^*p* < 0.001; ^##^*p* < 0.01, ^###^*p* < 0.001. *n* = 12 for mice in each group. The cell experiment was repeated three times independently.

Lastly, in order to clarify the effect of circRNA_0057209/miR-183/STK4 on thyroid tumorigenesis by influencing the Hippo pathway *via* inhibiting YAP *in vivo*, we injected nude mice with TPC-1 cells which had been stably transduced with lentivirus harboring oe-circRNA_0057209 or combined with lentivirus harboring sh-STK4. Subsequent results of qRT-PCR and Western blot analyses revealed that the expression levels of circRNA_0057209 and STK4 as well as YAP phosphorylation levels were elevated, while those of miR-183 were diminished in the TPC-1 cells manipulated with lentivirus harboring oe-circRNA_0057209. However, STK4 expression and YAP phosphorylation levels were found to be reduced in the TPC-1 cells manipulated with lentivirus harboring oe-circRNA_0057209 and sh-STK4 (Figure 8(a)). Meanwhile, IHC data showed that STK4 expression and YAP1 phosphorylation levels were markedly increased upon circRNA_0057209 overexpression, while silencing of STK4 reversed these alterations induced by circRNA_0057209 overexpression (Figure 8(b)). Additionally, the average tumor volume and weight of nude mice were found to be significantly reduced in response to circRNA_0057209 overexpression, and these effects could be negated by further STK4 silencing (Figures 8(c)–8(e)). Altogether, these findings indicated that the circRNA_0057209 prevented thyroid tumorigenesis by regulating the miR-183/STK4 signaling.

### 3.9. Discussion

**Figure 9:**
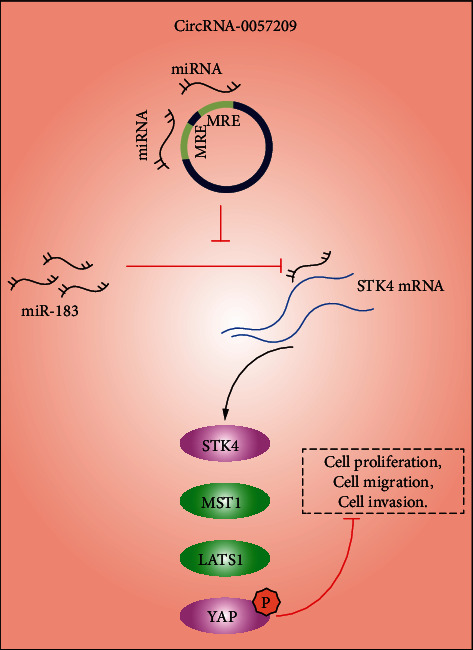
Schematic diagram of the mechanism by which circRNA_0057209 functioned in the thyroid cancer development. circRNA_0057209 upregulates STK4 through sponging miR-183 to activate the Hippo pathway, thereby inhibiting the proliferation, migration and invasion of thyroid cancer cells, and promoting cell apoptosis.

The advent of curative resection techniques and chemotherapy has greatly improved the overall survival of patients suffering from thyroid cancer [18]; however, it is prudent to advance the search for novel biomarkers to identify patients at risk of recurrence to facilitate regular and beneficial follow-ups [19]. Meanwhile, it is also important to recognize the hard-done work of our peers which has highlighted circRNAs as promising biomarkers due to their vital roles in the pathogenesis of cancer, the regulation of cell cycle, apoptosis, vascularization, and invasion, etc. [20]. In an effort to expand our knowledge of such, the current study set out to elaborate the significance of circRNA_0057209 in thyroid cancer and the mechanisms by which circRNA_0057209 participated in the pathogenesis of thyroid cancer. Consequently, we found that circRNA_0057209 acted as ceRNA of miR-183 and exerted a tumor-suppressive role in thyroid cancer by influencing the Hippo pathway *via* regulating the expression of STK4 (Figure 9).

Firstly, findings obtained in our study demonstrated that circRNA_0057209 was poorly-expressed in both thyroid cancer tissue and cell lines, whereas overexpression of circRNA_0057209 exerted inhibitory effects on the proliferation, migration, and invasion of thyroid cancer cells and a promotive effect on cell apoptosis. In addition, we discovered that circRNA_0057209 sequence contained a complementary binding sequence to miR-183, such that overexpression of circRNA_0057209 resulted in decreased expression of miR-183. The interaction between circRNAs and miRNAs is not a new discovery in the context of thyroid cancer progression; circRNA_0025033 was previously shown to interact with miR-1231 and miR-1304, leading to the promotion of papillary thyroid cancer cell proliferation and invasion [21]. Meanwhile, the miR-183 family is known as a highly conservative family, comprising of three members, namely, miR-96, miR-182, and miR-183 [22]. What is interesting is that a prior study documented upregulation of miR-183 in papillary thyroid carcinoma tissues and cell lines and that this upregulation augmented the manlignant potentials of cancer cells but inhibited their apoptosis [10]. Altogether, these findings and evidence make it plausible to suggest that circRNA_0057209 can delay the progression of thyroid cancer by inhibiting the expression of miR-183.

Another topic of focus in our study, the mammalian Hippo pathway is known to be composed of mammalian Ste20-like kinases (MST1/2) and large tumor suppressor kinases (LATS1/2) that possess the ability to regulate transcriptional coactivators such as YAP1 and TAZ [23]. The aforementioned YAP1 is capable of influencing macrophages, myeloid-derived suppressor cells, and regulatory T-cells, in order to facilitate immunosuppressive tumor microenvironment by activating pathways in various cellular components [24]. It is also noteworthy that YAP1 is highly expressed in thyroid cancer, while silencing of YAP1 was previously shown to suppress the cell proliferation *via* mediation of the PI3K-Akt pathway [25]. As a result, it would be sapient to explore interaction of YAP1, circRNAs, and miRNAs, with various studies already uncovering certain correlations amongst them. For instance, YAP1 promotes oncogenic activities of miR-17 and miR-106b by transcriptionally-inhibiting the expression of circRNA_000425 [26]. Similarly, a prior study has illustrated that miR-205 inhibits malignant cancer progression in thyroid cancer, whereas upregulation of YAP1 partially restrains the antitumor role of miR-205 [27]. The current study failed to explore the direct relation between YAP1 and miR-183 or circRNA_0057209 in the progression of thyroid cancer, and thus requires further comprehensive studies in the future. Nevertheless, our findings revealed that STK4 positively regulated the expressions of MST1, LAST1, and LAST2 and the phosphorylation levels of YAP, thus modulating the Hippo pathway, wherein STK4 was controlled and mediated by miR-183 and circRNA_0057209, which collectively provide a novel insight into the mechanism by which circRNA_0057209 and miR-183 mediate the Hippo pathway in thyroid cancer.

Additionally, a prior study indicated that STK4 deficiency can precipitate primary immunodeficiency, which is associated with lymphomas [28]. Inherently, STK4 is known as a key component of the Hippo tumor suppressor pathway, while the ablation of STK4 is associated with progression of hepatocellular carcinoma [29]. Meanwhile, our findings revealed that STK4 is poorly expressed in thyroid cancer tissues and cell lines, while silencing of STK4 could strengthen the malignancy of thyroid cancer cells. Interestingly, we also found that treatment with verteporfin, a YAP1 inhibitor, abrogated the effect of STK4 silencing on thyroid cancer cells. Moreover, it has been described that overexpression of STK4, knockdown of YAP, or verteporfin treatment can inhibit the proliferation in natural killer T-cell lymphoma cells, whereas knockdown of STK4 and over-expression of YAP bring about augmented lymphoma cell proliferation [30]. Further in agree with our findings, another prior study indicated that STK4 was reduced in thyroid cancer, whereas upregulation of STK4 not only impeded the proliferation and resistance to apoptosis but also augmented the autophagy in thyroid cancer through the Hippo pathway [17]. Collectively, these findings and data demonstrate that STK4 restrains the malignant progression of thyroid cancer *via* regulating the Hippo pathway.

## 4. Conclusions

Altogether, findings obtained in our study indicated that circRNA_0057209 can potentially weaken the miR-183 binding ability to STK4 and activate the Hippo pathway, thus inhibiting the malignant development of thyroid cancer (Figure 9). Our study sheds a new light on the functional mechanism of circRNA_0057209 in thyroid cancer which can lay the foundation stone for promising therapeutic targets against thyroid cancer. Nevertheless, future experiments are warranted to validate these findings and expand the translational potential in this direction, aiming to improve the quality of life of patients plagued by thyroid cancer.

## Data Availability

The data and materials of the study can be obtained from the corresponding author upon request.

## Supplementary Materials

Supplementary Materials Supplementary Figure 1: schematic diagram of the sequence and structure of circRNA_0057209 in thyroid cancer and the expression of circRNA_0057209 and linear TTN-AS1-RNA in thyroid cancer cells. (A) Schematic diagram of primer design site. (B) Schematic diagram of circRNA_0057209 structure and connection sequence in thyroid cancer. Red indicates exon11 of TTN-AS1, purple indicates exon2, and the position indicated by the arrow refers to the junction site of the two. (C) Blast results in NCBI after amplification of the junction site sequence with circ-F/R primers. (d) The expression of circRNA_0057209 and linear TTN-AS1-RNA in TPC-1 and HTH-83 cells determined by qRT-PCR. ^∗∗∗^*p* < 0.001. The cell experiment was repeated three times independently. Supplementary Figure 2: representative images of Transwell migration and invasion assays. (A) Representative images of Transwell assay showing the migration of oe-circRNA_0057209-treated TPC-1 and HTH83 cells. (B) Representative images of Transwell assay showing the invasion of oe-circRNA_0057209-treated TPC-1 and HTH83 cells. (C) Representative images of Transwell assay showing thyroid cancer cell migration after treatment with oe-circRNA_0057209 or combined with miR-183 mimic. (D) Representative images of Transwell assay showing cell invasion after treatment with oe-circRNA_0057209 or combined with miR-183 mimic. Supplementary Table 1: the specific grouping of the samples in the GSE93522 and GSE40807 expression datasets. Supplementary Table 2: qRT-PCR primer sequences. Supplementary Table 3: correlation between circRNA_0057209 expression and clinicopathological characteristics of thyroid cancer patients.
