# Supplementary material for: Circular RNA_0057209 Acts as ceRNA to Inhibit Thyroid Cancer Progression by Promoting the STK4-Mediated Hippo Pathway via Sponging MicroRNA-183
Source: Oxid Med Cell Longev. 2022 Mar 11;2022:9974639. doi: 10.1155/2022/9974639 (PMC8933075; doi:10.1155/2022/9974639)
Supplement: Supplementary Materials — Supplementary Figure 1: schematic diagram of the sequence and structure of circRNA_0057209 in thyroid cancer and the expression of circRNA_0057209 and linear TTN-AS1-RNA in thyroid cancer cells. (A) Schematic diagram of primer design site. (B) Schematic diagram of circRNA_0057209 structure and connection sequence in thyroid cancer. Red indicates exon11 of TTN-AS1, purple indicates exon2, and the position indicated by the arrow refers to the junction site of the two. (C) Blast results in NCBI after amplification of the junction site sequence with circ-F/R primers. (d) The expression of circRNA_0057209 and linear TTN-AS1-RNA in TPC-1 and HTH-83 cells determined by qRT-PCR. ∗∗∗p < 0.001. The cell experiment was repeated three times independently. Supplementary Figure 2: representative images of Transwell migration and invasion assays. (A) Representative images of Transwell assay showing the migration of oe-circRNA_0057209-treated TPC-1 and HTH83 cells. (B) Representative images of Transwell assay showing the invasion of oe-circRNA_0057209-treated TPC-1 and HTH83 cells. (C) Representative images of Transwell assay showing thyroid cancer cell migration after treatment with oe-circRNA_0057209 or combined with miR-183 mimic. (D) Representative images of Transwell assay showing cell invasion after treatment with oe-circRNA_0057209 or combined with miR-183 mimic. Supplementary Table 1: the specific grouping of the samples in the GSE93522 and GSE40807 expression datasets. Supplementary Table 2: qRT-PCR primer sequences. Supplementary Table 3: correlation between circRNA_0057209 expression and clinicopathological characteristics of thyroid cancer patients. [file 9974639.f1.docx]

**
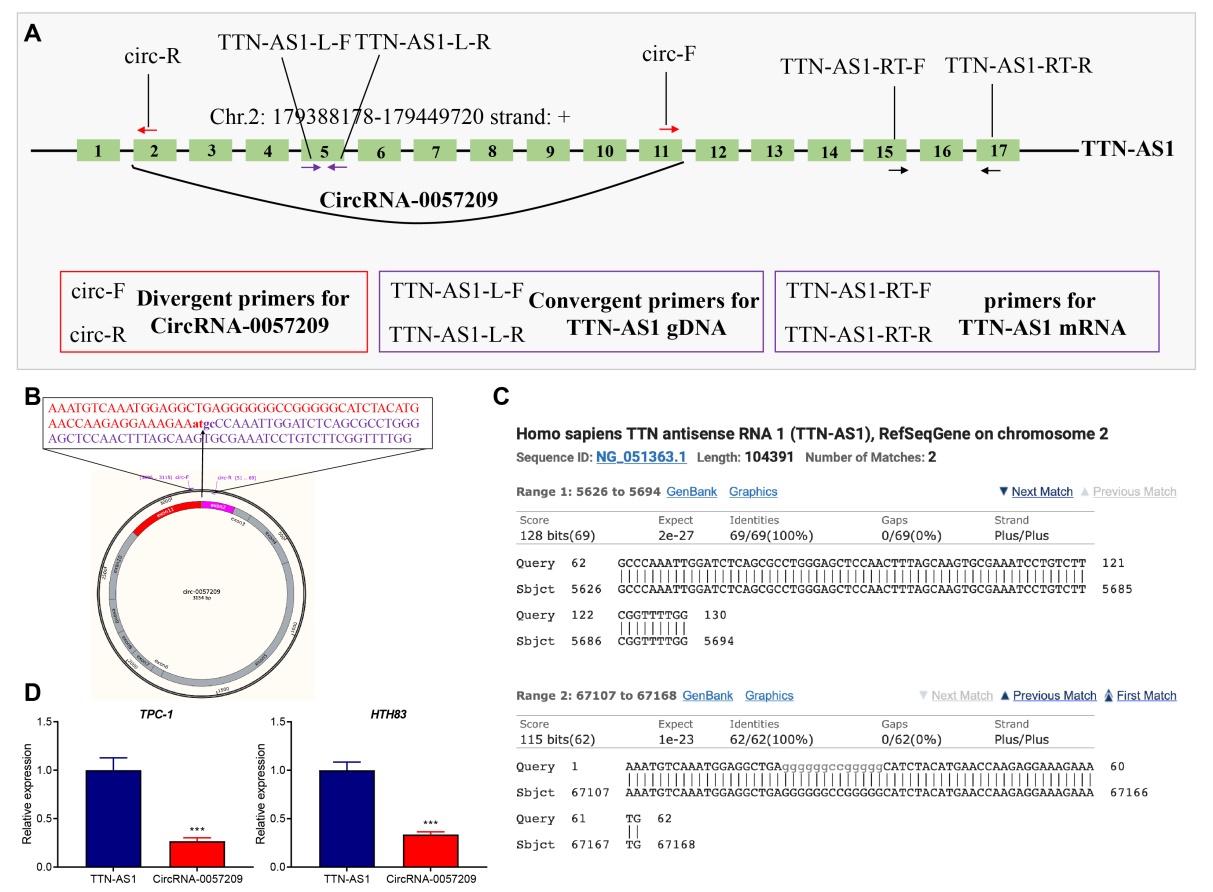
**

**Supplementary Figure 1:** Schematic diagram of the sequence and structure of circRNA_0057209 in thyroid cancer and the expression of circRNA_0057209 and linear TTN-AS1-RNA in thyroid cancer cells. A: Schematic diagram of primer design site. B: Schematic diagram of circRNA_0057209 structure and connection sequence in thyroid cancer. Red indicates exon11 of TTN-AS1, purple indicates exon2, and the position indicated by the arrow refers to the junction site of the two. C: Blast results in NCBI after amplification of the junction site sequence with circ-F/R primers. D: the expression of circRNA_0057209 and linear TTN-AS1-RNA in TPC-1 and HTH-83 cells determined by qRT-PCR. *** *p* < 0.001. The cell experiment was repeated three times independently.

**
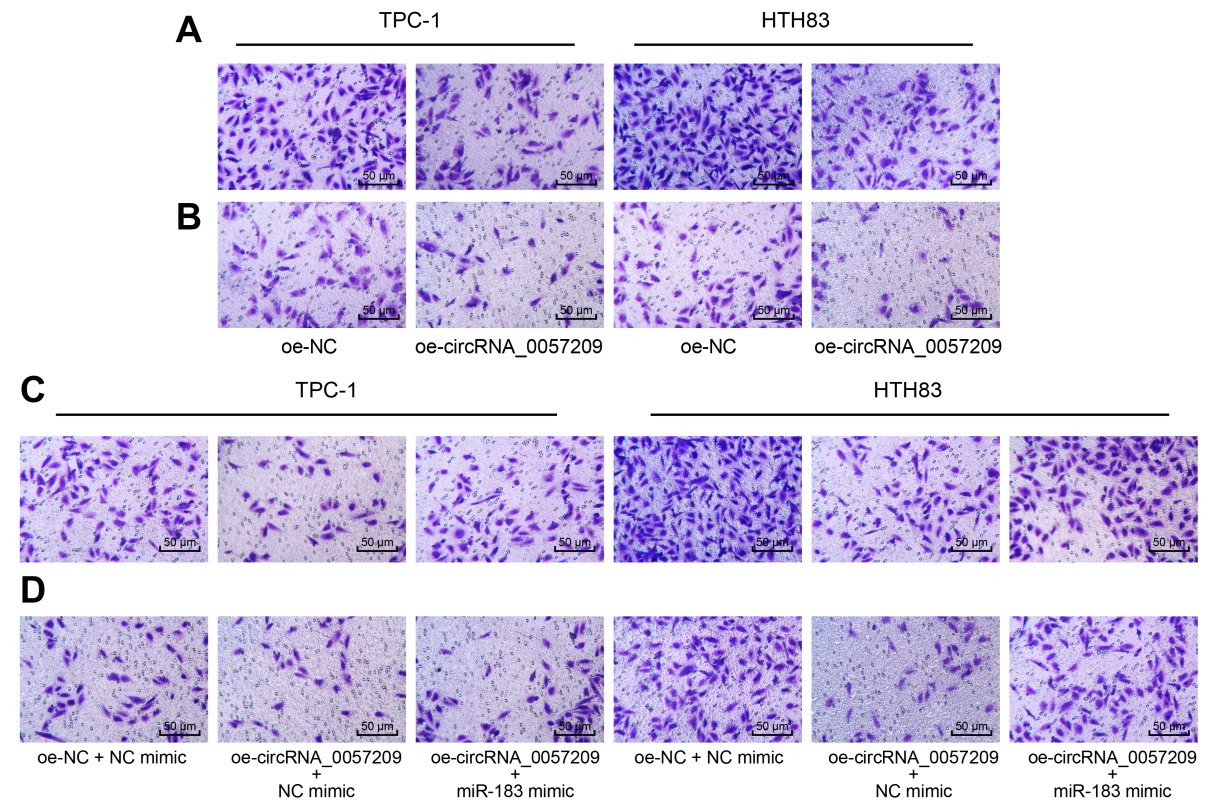
**

**Supplementary Figure 2:** Representative images of Transwell migration and invasion assays. A: Representative images of Transwell assay showing the migration of oe-circRNA_0057209-treated TPC-1 and HTH83 cells. B: Representative images of Transwell assay showing the invasion of oe-circRNA_0057209-treated TPC-1 and HTH83 cells. C: Representative images of Transwell assay showing thyroid cancer cell migration after treatment with oe-circRNA_0057209 or combined with miR-183 mimic. D: Representative images of Transwell assay showing cell invasion after treatment with oe-circRNA_0057209 or combined with miR-183 mimic.

**Supplementary Table 1** The specific grouping of the samples in the GSE93522 and GSE40807 expression datasets

| GEO accession | Normal samples | Tumor samples | Platform |
| --- | --- | --- | --- |
| GSE93522 | 6 | 6 | GPL19978 |
| GSE40807 | 40 | 40 | GPL8227 |

**Supplementary Table 2** qRT-PCR primer sequences

| Target | Primer sequence |
| --- | --- |
| circRNA_0057209 | circ-F: 5'-ATGTCAAATGGAGGCTGAGG-3' |
|  | circ-R: 5'-CCAAAACCGAAGACAGGAT-3' |
| TTN-AS1-gDNA | TTN-AS1-L-F: 5'-TGGTTGAAAGGGCACTTACTCA-3' |
|  | TTN-AS1-L-R: 5'-GTCCCAACAGTCCAGAAGGG-3' |
| TTN-AS1 | TTN-AS1-RT-F: 5'-CCTGCGAGACTGCGGAATAA-3' |
|  | TTN-AS1-RT-R: 5'-TTCCTTTCCAGACCGCCCAT-3' |
| miR-183 | F: 5'-AGAGTGTGACTCCTGTTCTG-3'  R: 5'-CTGTCTCTGCTCTGTTTATGG-3' |
| STK4 | F: 5'-GGTCAAGATTGCTGAGTGAGTG-3' |
|  | R: 5'-TCACAGATGGAGAGCCGAGT-3' |
| U6 | F: 5'-CTCGCTTCGGCAGCACA-3' |
|  | R: 5'-AACGCTTCACGAATTTGCGT-3' |
| GAPDH-convergent | Con-F: 5'-GCACCGTCAAGGCTGAGAAC-3'  Con-R: 5'-TGGTGAAGACGCCAGTGGA-3' |
| GAPDH-divergent | DIV-F: 5'-GCGACACCCACTCCTCCAC-3' |
|  | DIV-R: 5'-TGGCAACAATATCCACTTTACC-3' |

Notes: F, forward, R, reverse, Con-F, Convergent forward, Con-R, Convergent reverse, DIV-F, Divergent forward, DIV-R, Divergent reverse

**Supplementary Table 3** Correlation between circRNA_0057209 expression and clinicopathological characteristics of thyroid cancer patients

| Clinicopathological features | Case (n = 68) | circRNA_0057209 expression | | *p* value |
| --- | --- | --- | --- | --- |
|  |  | High | Low |  |
| Age (years) |  |  |  | 0.808 |
| > 50 | 31 | 16 | 15 |  |
| ≤ 50 | 37 | 18 | 19 |  |
| Gender |  |  |  | 0.301 |
| Male | 22 | 9 | 13 |  |
| Female | 46 | 25 | 21 |  |
| Tumor size (cm) |  |  |  | 0.0009 |
| ≥ 3 | 45 | 16 | 29 |  |
| < 3 | 23 | 18 | 5 |  |
| TNM stage |  |  |  | < 0.0001 |
| I-II | 38 | 27 | 11 |  |
| III + Ⅳ | 30 | 7 | 23 |  |
| Lymph node metastasis |  |  |  | < 0.0001 |
| Yes | 28 | 5 | 23 |  |
| No | 40 | 29 | 11 |  |
| Differentiation |  |  |  | 0.0002 |
| Well and moderate | 43 | 29 | 14 |  |
| Poor | 25 | 5 | 20 |  |
